# Pembrolizumab and Nivolumab-Induced Late Drug Reaction With Eosinophilia and Systemic Symptoms (DRESS): A Case Report

**DOI:** 10.7759/cureus.104469

**Published:** 2026-03-01

**Authors:** Ahmed Alqefari, Abdulelah A Aldossari, Mohammed Y Almohaimeed, Khalid A Aldossari, Ayoub Almohaimeed, Naif Aldossari, Hala Elsaka

**Affiliations:** 1 College of Medicine, Qassim University, Buraydah, SAU; 2 Dermatology, King Fahad Specialist Hospital, Buraydah, SAU; 3 Community and Family Medicine, King Saud University, Riyadh, SAU; 4 Family Medicine, The Riyadh Second Health Cluster, Riyadh, SAU; 5 Pathology, Faculty of Medicine, Ain Shams University, Cairo, EGY; 6 Pathology, King Fahad Specialty Hospital, Dammam, SAU

**Keywords:** drug reaction with eosinophilia and systemic symptoms (dress), immune checkpoint inhibitors (icis), immune-related adverse events (iraes), nivolumab, pembrolizumab

## Abstract

Drug reaction with eosinophilia and systemic symptoms (DRESS) is a rare, potentially life-threatening immune-related adverse event that can be triggered by immune checkpoint inhibitors. We present the case of a 58-year-old woman with malignant acral melanoma who developed late-onset DRESS following sequential treatment with pembrolizumab and nivolumab. The patient initially developed a generalized erythrodermic, scaly rash with pruritus after the third dose of pembrolizumab, which recurred upon switching to nivolumab. Laboratory evaluation revealed leukocytosis, marked eosinophilia, transaminitis, and elevated lactate dehydrogenase. Skin biopsies demonstrated spongiosis, basal vacuolar degeneration, eosinophilic infiltrate, and leukocytoclastic vasculitis, consistent with DRESS. Despite corticosteroid therapy, the patient experienced multiple relapses triggered by immunosuppressant tapering, requiring prolonged cyclosporine treatment. Her presentation met the registry of severe cutaneous adverse reactions (RegiSCAR) criteria for definite DRESS. This case underscores the need for heightened awareness of delayed hypersensitivity reactions to immune checkpoint inhibitors and highlights the possibility of a chronic-relapsing course requiring extended immunosuppressive therapy.

## Introduction

The advent of immune checkpoint inhibitors (ICIs) has reshaped oncology, offering unprecedented survival benefits for advanced malignancies, such as melanoma and squamous cell carcinoma [1,2]. Agents such as pembrolizumab and nivolumab, monoclonal antibodies targeting the programmed cell death protein-1/programmed death-ligand 1 (PD-1/PD-L1)pathway, work by unleashing T-cell-mediated antitumor immunity [2,3]. However, this breakthrough comes with a trade-off: immune-related adverse events (IRAEs) that frequently target the skin, ranging from mild rashes to life-threatening conditions such as bullous pemphigoid [3,4]. Among the rarest and most treacherous IRAEs are drug reactions with eosinophilia and systemic symptoms (DRESS). Characterized by fever, diffuse rash, eosinophilia, and multiorgan involvement (hepatic, renal, or pulmonary), DRESS carries a mortality risk of up to 10%. Historically linked to anticonvulsants or antibiotics, DRESS is increasingly recognized as an ICI complication [2]. We present a novel case of late DRESS syndrome following sequential pembrolizumab and nivolumab therapy. This report underscores the diagnostic conundrum posed by delayed hypersensitivity reactions to ICIs and highlights the critical importance of vigilance in patients receiving prolonged immunotherapy.

## Case presentation

**Figure 1:**
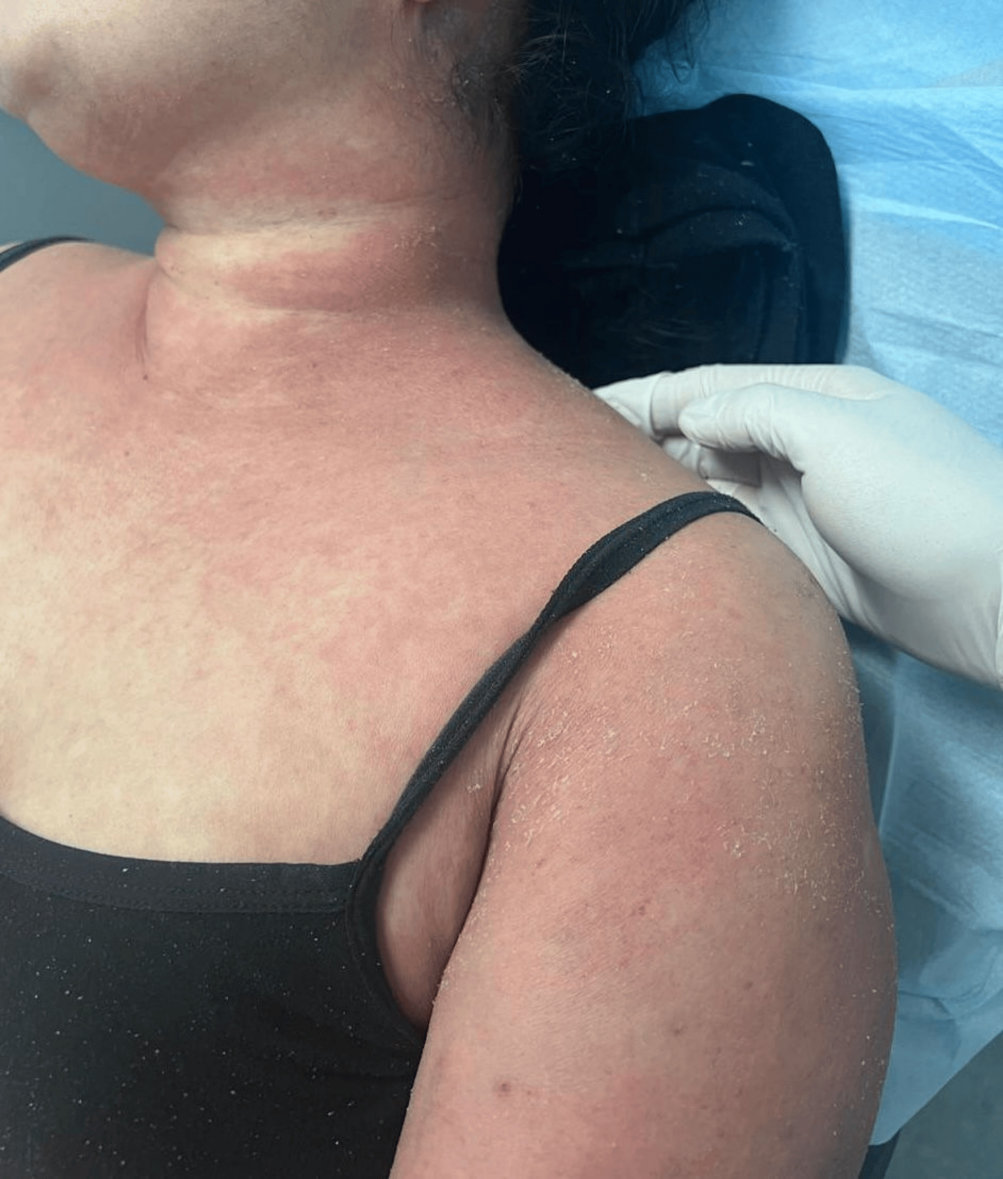
Confluent erythematous patches with fine scaling over the neck and upper trunk and accentuation within skin folds

A 58-year-old Indonesian woman with a history of malignant acral melanoma (pathologic stage pT3b, post-resection) presented with a progressive, pruritic skin eruption during the course of adjuvant immunotherapy. She had received three cycles of pembrolizumab (200 mg IV every three weeks) as part of a planned treatment regimen when the rash developed. Approximately 10 days after the third pembrolizumab infusion, she developed a generalized erythrodermic, scaly, morbilliform rash sparing the face (Figure 1). Similar but milder transient rashes had occurred after her earlier cycles. On examination, she was afebrile, hemodynamically stable, and had no mucosal involvement, genital lesions, lymphadenopathy, or positive Nikolsky sign. The dermatology team's initial impression was a pembrolizumab-induced eczematous drug reaction, and management included an oral prednisolone taper starting at 40 mg daily and decreasing to 5 mg over 30 days, along with cetirizine and topical mometasone for symptomatic relief.

**Figure 2:**
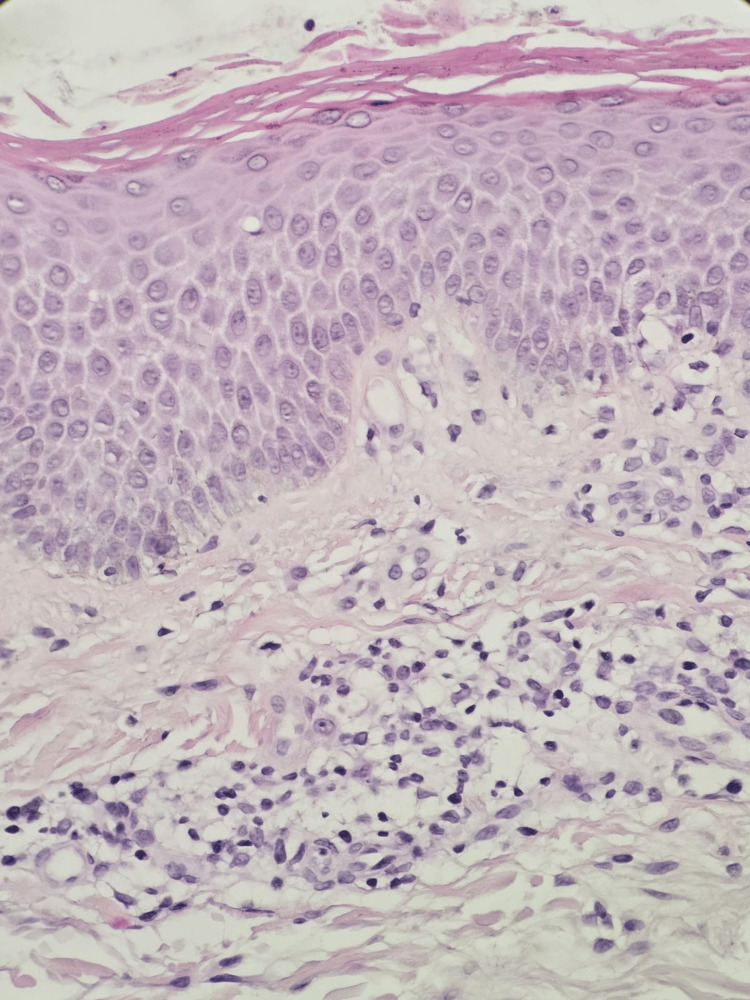
Dermal perivascular mixed inflammatory cell infiltrate with eosinophils (H&E stain)

**Figure 3:**
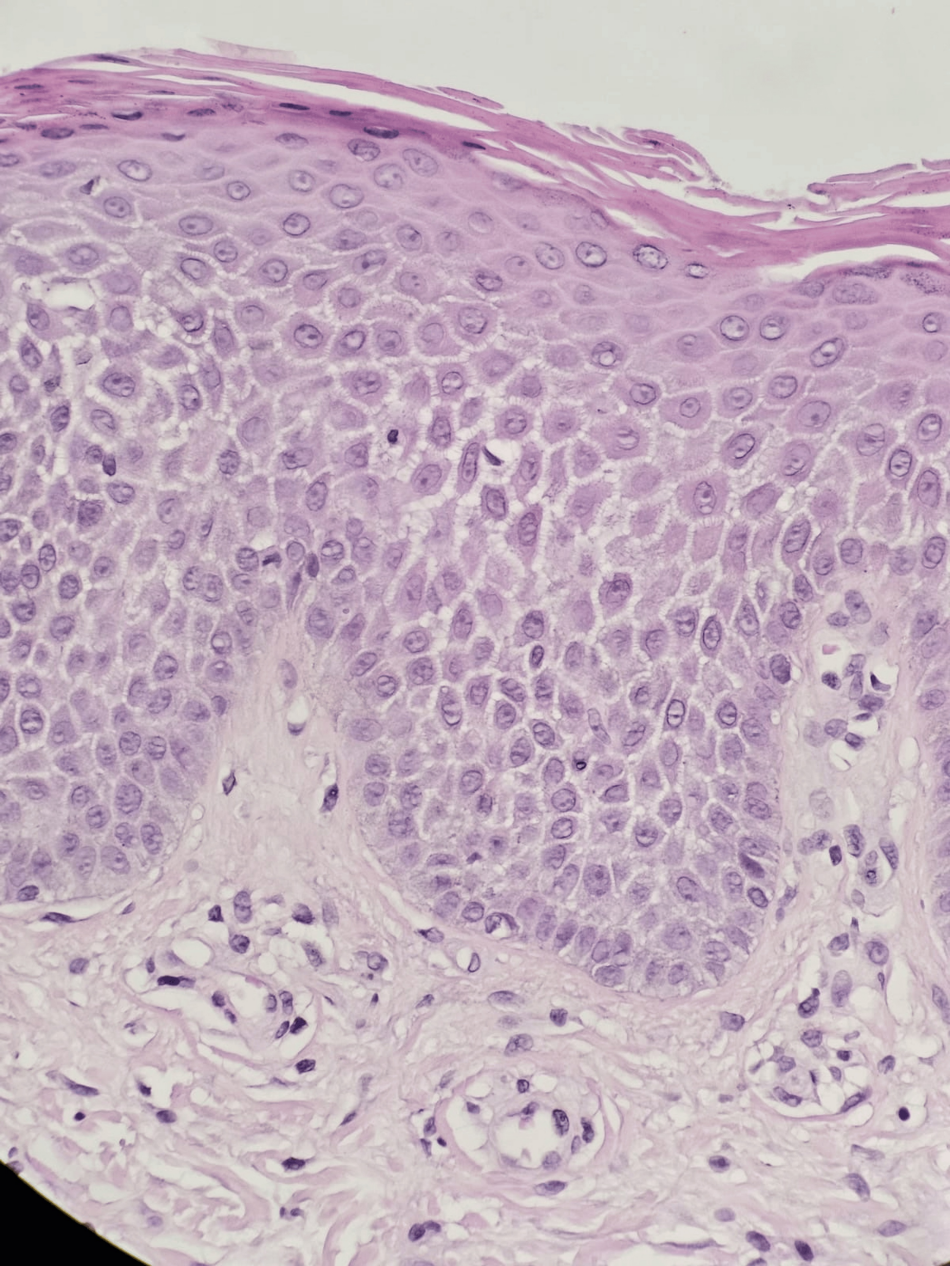
Epidermis showing hyperkeratosis, acanthosis, and spongiosis (H&E stain)

**Figure 4:**
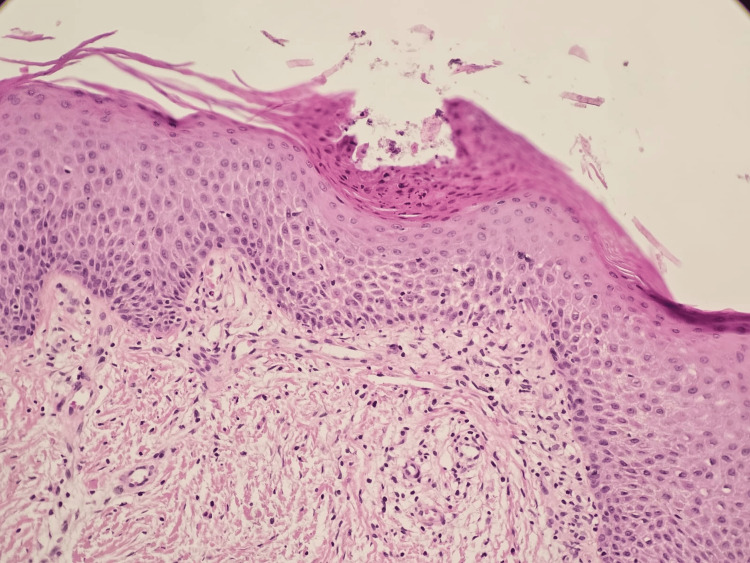
Epidermis with hyperkeratosis, parakeratosis, acanthosis, and Munro's microabscess (H&E stain)

Following resolution of the initial rash, her treatment was switched to nivolumab (240 mg IV every two weeks). However, within two weeks of starting nivolumab, she experienced recurrence of the same rash. Initial skin biopsy from the abdomen revealed spongiosis and vacuolar interface dermatitis with eosinophilic infiltrate, suggestive of DRESS and lichenoid reaction (Figures 2-4).

**Figure 5:**
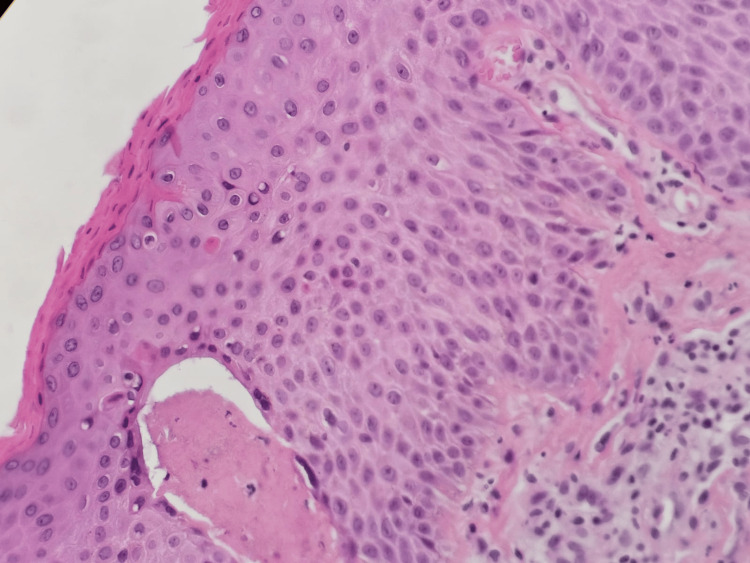
Epidermis with acanthosis, scattered apoptotic keratinocytes, and focal basal cell vacuolar change (H&E stain)

**Figure 6:**
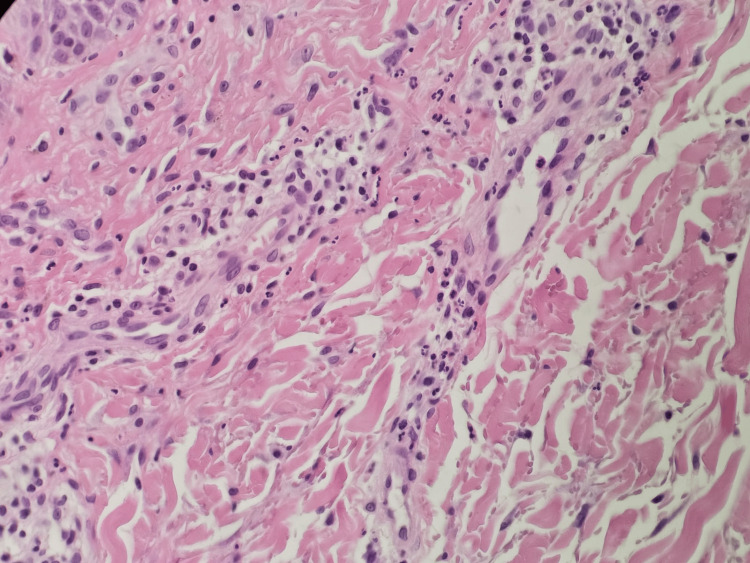
Dermal blood vessels demonstrating prominent endothelial lining and leukocytoclastic vasculitis (H&E stain)

Subsequent biopsies from the abdomen and thigh demonstrated more characteristic features, including leukocytoclastic vasculitis and persistent eosinophilic infiltrates, supporting the diagnosis of DRESS (Figures 5-6).

Special stains excluded infectious organisms, and immunohistochemical staining ruled out malignancy. Laboratory investigations (Table 1) revealed leukocytosis (peak 16.43×10³/µL) with eosinophilia (peak 1.8×10³/µL), elevated lactate dehydrogenase (LDH, 566 U/L), and thrombocytosis (platelets 486×10³/µL) during flares. Despite initial response to corticosteroids, she experienced recurrent flares whenever immunosuppression was tapered, ultimately requiring the addition of cyclosporine (100 mg once or twice daily) for steroid-refractory disease. Over the following months, she continued to exhibit a relapsing course characterized by pruritic lichenified acral plaques, desquamation, hair thinning, and recurring laboratory abnormalities, including eosinophilia and elevated LDH. The clinical picture was consistent with DRESS, meeting the registry of severe cutaneous adverse reactions (RegiSCAR) diagnostic criteria: acute rash, eosinophilia, systemic involvement (hepatitis and hematologic abnormalities), and suspected drug causality from PD-1 inhibitors. Her RegiSCAR score was 7, fulfilling the criteria for a definite case of DRESS (Table 2).

**Table 1:** Laboratory findings "Verified 2" indicates that the reported laboratory values were reviewed and confirmed by a second-level laboratory verification process in accordance with institutional quality control procedures. MPV - mean platelet volume; APTT - activated partial thromboplastin time; INR - international normalized ratio

Test	Result ^(verified 2)^	Reference range
ALT (alanine aminotransferase)	10 U/L	7-56
AST (aspartate aminotransferase)	16.5 U/L	5-34
Albumin	29.52 g/L	32-46
Blood group	A positive	N/A
Neutrophils	6.3 ×10³/µL	1-6.5
Lymphocytes	1.9 ×10³/µL	1.3-3.8
Monocytes	0.7 ×10³/µL	0.2-0.9
Eosinophil	1.1 ×10³/µL	0-0.4
Basophils	0.1 %	0-0.1
MPV	6.9 fL	9.2-12.7
Neutrophil	62.50 %	44.6-58.6
APTT	31.2 s	24-40
INR	0.88 x10^3^/uL	0.9-1.3
Prothrombin time	12.4 s	10.5-15.8

**Table 2:** RegiSCAR scoring for the patient DRESS - drug reaction with eosinophilia and systemic symptoms; RegiSCAR - the registry of severe cutaneous adverse reactions

Criterion	Findings in the patient	Score
Fever ≥38.5°C	No documented fever; afebrile throughout	-1
Enlarged lymph nodes (≥2 sites, >1 cm, tender)	No lymphadenopathy noted	0
Involvement of internal organ(s)	Liver involvement (transaminitis reported during flares)	1
Blood eosinophilia	1.8 ×10⁹/L → ≥1.5 ×10⁹/L	2
Atypical lymphocytes	Not reported	0
Skin involvement >50% body surface area (BSA)	Generalized erythrodermic rash	1
Skin rash suggestive of DRESS (e.g., infiltrated, facial edema, purpura)	Scaly, morbilliform rash with acral involvement; pruritus and lichenification later	1
Skin biopsy suggesting DRESS	Interface dermatitis with spongiosis and eosinophils; supportive of DRESS in the appropriate clinical context	1
Rash resolution >15 days	Chronic-relapsing course over eight months	1
Evaluation to exclude other causes (e.g., infection, malignancy)	Infections and malignancy were ruled out by histology and serology	1
Total RegiSCAR score		7

## Discussion

DRESS is a rare but potentially life-threatening immune-mediated hypersensitivity syndrome. It is classically characterized by fever, a widespread rash, eosinophilia, and internal organ involvement, most commonly affecting the liver, kidneys, or lungs [4]. While ICIs are known for various dermatologic adverse effects, DRESS remains among the rarest of these toxicities [5]. Diagnosis hinges on clinical and laboratory findings, including those outlined in the RegiSCAR criteria [6]: delayed rash, hematologic abnormalities (such as eosinophilia), and internal organ involvement [7]. Although initially developed for conventional drugs, RegiSCAR has become increasingly applicable to ICI-associated cases. In our patient, late-onset symptoms, relapses upon immunosuppressant tapering, and cross-reactivity between pembrolizumab and nivolumab strongly supported a class-wide hypersensitivity response. Management typically requires prompt discontinuation of the offending agent and initiation of high-dose corticosteroids. For patients with steroid-refractory or relapsing disease, immunosuppressants such as cyclosporine may be necessary [3]. In this case, oral cyclosporine successfully controlled subsequent flares, consistent with its use in other reports of severe or relapsing DRESS.

Our case adds to the limited literature by demonstrating a chronic-relapsing phenotype of DRESS following sequential PD-1 inhibitor therapy. To date, only four published cases of PD-1 inhibitor-induced DRESS have been identified globally [1,5,8,9], and none have documented the chronic-relapsing pattern observed in this patient. Like our patient, Tran et al. reported pembrolizumab-induced DRESS with delayed onset, though their case lacked the hepatic dysfunction seen here [1]. This underscores the variability in organ involvement despite shared triggers. Lu et al. documented nivolumab-induced DRESS with a rapid onset [8], mirroring our patient's progression upon switching to nivolumab. Mirza et al.'s biopsy findings, like in our case, showed vacuolar interface dermatitis with eosinophils, reinforcing DRESS as a histopathologic mimic of other drug eruptions. Their case, however, resolved faster without chronic relapses [10]. Moreover, their biopsy findings were similar to ours, vacuolar interface dermatitis with eosinophils, highlighting the histological overlap between DRESS and other drug eruptions [10]. Sun et al. successfully used cyclosporine for steroid-refractory DRESS, akin to our approach [3]. This supports cyclosporine as a salvage therapy for ICI-related cases, though optimal duration remains unclear. Dao et al. reported pembrolizumab-induced hyper-eosinophilia with prolonged symptoms, akin to our patient's relapsing course [11]. Both cases challenge the paradigm of DRESS as self-limiting, suggesting ICIs may trigger persistent immune dysregulation. The shared eosinophilic infiltrates across biopsies [8,10] suggest T-cell-mediated hypersensitivity as a unifying pathway. However, the delayed onset in our case hints at cumulative immune dysregulation.

This case not only expands the clinical spectrum of ICI-related hypersensitivity but also emphasizes the importance of long-term follow-up, viral monitoring, and dermatologic consultation for persistent or atypical rashes. Given the widespread and increasing use of ICIs, clinicians must maintain a high index of suspicion for late and prolonged immune-related adverse events. Our experience reinforces the need for applying standardized diagnostic tools, such as RegiSCAR, and considering extended immunosuppression in patients with relapsing disease courses.

## Conclusions

This case highlights a rare but clinically significant example of late-onset, chronic-relapsing DRESS triggered by sequential PD-1 inhibitor therapy with pembrolizumab and nivolumab. It underscores the expanding spectrum of immune-related adverse events associated with immune checkpoint inhibitors and the importance of recognizing delayed hypersensitivity reactions even after discontinuation or switching of agents within the same drug class. The chronic, relapsing nature of the disease in this patient also emphasizes the need for prolonged immunosuppressive therapy and close follow-up. Dermatologic vigilance and application of diagnostic tools, such as the RegiSCAR scoring system, are essential to ensure timely diagnosis and optimal management. This case adds to the limited literature on ICI-induced DRESS and serves as a reminder of the complex immune dynamics involved in cancer immunotherapy.
